# Absorbable collagen sponges loaded with recombinant bone morphogenetic protein 9 induces greater osteoblast differentiation when compared to bone morphogenetic protein 2

**DOI:** 10.1002/cre2.55

**Published:** 2017-02-09

**Authors:** Masako Fujioka‐Kobayashi, Benoit Schaller, Nikola Saulacic, Benjamin E. Pippenger, Yufeng Zhang, Richard J. Miron

**Affiliations:** ^1^ Department of Periodontology, College of Dental Medicine Nova Southeastern University Florida USA; ^2^ Department of Cranio‐Maxillofacial Surgery, Bern University Hospital Inselspital Switzerland; ^3^ Department of Oral Surgery, Clinical Dentistry, Institute of Biomedical Sciences Tokushima University Graduate School Japan; ^4^ Institut Straumann AG Surgical and Clinical Science Research Department Switzerland; ^5^ Department of Oral Implantology University of Wuhan China; ^6^ Department of Periodontics and Oral Medicine University of Michigan School of Dentistry Michigan USA

**Keywords:** BMP2, BMP9, bone formation, GBR, guided bone regeneration, osteoinductive

## Abstract

The use of growth factors for the regeneration of soft and hard tissues has been utilized extensively in dental medicine over the past decade. Recently our group found that recombinant human bone morphogenetic protein 9 (rhBMP9) was more osteopromotive than recombinant human bone morphogenetic protein 2 (rhBMP2) when combined with a deprotenized bovine bone mineral bone grafting material. The aim of the present in vitro study was to evaluate the regenerative potential of an absorbable collagen sponge(ACS) specifically designed for extraction socket healing loaded with rhBMP9 when compared to rhBMP2. The adsorption and release kinetics of rhBMP2 and rhBMP9 were first investigated by enzyme‐linked immunosorbent assay quantification. Then, the cellular effects of stromal cell line (ST2) preosteoblasts were investigated utilizing four groups including rhBMP2 and rhBMP9 at both low(10 ng/ml) and high(100 ng/ml) concentrations loaded onto ACS. Cellular attachment(8 hours) and proliferation(1, 3, and 5 days) as well as osteoblast differentiation were investigated by real‐time polymerase chain reaction (PCR) at 3 and 14 days, alkaline phosphatase (ALP) activity at 7 days, and alizarin red staining at 14 days. ACS fully adsorbed both rhBMP2 and rhBMP9 that were slowly released up to 10 days. Although neither rhBMP2 nor rhBMP9 had any effects on cell attachment or proliferation, pronounced effects were observed on osteoblast differentiation. ALP activity was increased seven‐fold with rhBMP2‐high, whereas a marked 10‐fold and 20‐fold increase was observed with rhBMP9‐low and high loaded to ACS, respectively. Furthermore, mRNA levels of collagen1, ALP, bone sialoprotein, and osteocalcin were all significantly higher for rhBMP9 when compared to control or rhBMP2 groups. Alizarin red staining further confirmed that rhBMP9‐low and high demonstrated marked increases in mineralization potential when compared to rhBMP2‐high. The results demonstrate the marked effect of rhBMP9 on osteoblast differentiation when combined with ACS in comparison to rhBMP2 at doses as much as 10 times lower. Further in vivo studies are necessary to investigate whether the regenerative potential is equally as potent.

## 1 INTRODUCTION

Growth factors have played a pivotal role in modern dentistry for the regeneration of periodontal and/or bone defects caused by trauma, periodontal disease, congenital abnormalities, and tumors (Alonso et al., 2010; Behnia, Khojasteh, Soleimani, Tehranchi, & Atashi, 2012; Cicciu, Herford, Cicciu, Tandon, & Maiorana, 2014; Herford, Tandon, Stevens, Stoffella, & Cicciu, 2013; Ramseier, Rasperini, Batia, & Giannobile, 2012; Yokota et al., 2014). Examples of these include the use of platelet‐derived growth factor for periodontal and peri‐implant defects (Kaigler et al., 2011), enamel matrix derivative for periodontal intrabony defects (Miron et al., 2016), and fibroblast growth factor two for periodontal defects (Cochran et al., 2016). One growth factor that has received widespread use for the regeneration of pure bone defect is that of bone morphogenetic protein 2 (BMP2) utilized across many fields of medicine (Carreira et al., 2015; Lin, Lim, Chan, Giannobile, & Wang, 2015; Schroeder et al., 2016).

In general, BMPs are important pleiotropic proteins playing a pivotal role in the commitment and differentiation of osteoprogenitor cells toward bone forming osteoblasts (Rosen, 2006). Although recombinant human (rh)BMP2 is the most widely utilized recombinant BMP with Food and Drug Administration (FDA)'s approval (Bessa, Casal, & Reis, 2008a, 2008b; Carreira et al., 2014), many investigators remain surprised to learn that other BMPs have previously been characterized as more osteogenic than the currently approved BMP2 or BMP7 (Kang et al., 2004). In two pioneering study conducted over a decade ago investigating all 14 BMPs, it was found that BMP6 and BMP9 stimulated the highest alkaline phosphatase (ALP) expression in vitro (Cheng et al., 2003) and had greater potential for orthotropic ossification in vivo (Kang et al., 2004). In those studies, the bone‐inducing properties of all BMPs were reported using adenovirus transfection experiments (gene therapy), an area of research still not approved by the FDA for clinical use (Balmayor & van Griensven, 2015).

BMP9 (also known as growth differentiation factor 2; GDF2) was first identified in the developing mouse liver cDNA libraries (Song et al., 1995) and has since been characterized as the most osteogenetic BMP showing more bone‐regenerative potential than BMP2, also acting as a major modulator of angiogenesis and chondrogenesis (Blunk et al., 2003; Lamplot et al., 2013; Leblanc et al., 2011). Although these previous studies were confirmed only utilizing BMP9‐adenovirus‐transfected cells not approved by the FDA (Cheng et al., 2003; Kang et al., 2004; Lamplot et al., 2013; Leblanc et al., 2011), our research group recently characterized the regenerative potential of rhBMP9 in comparison to rhBMP2. In two studies, it was found that rhBMP9 demonstrated up to 10 times greater osteogenic differentiation when compared to rhBMP2 (Fujioka‐Kobayashi et al., 2016a, 2016b). These prominent findings bring intriguing new possibilities for future clinical application for the treatment of large bone defects in either medicine or dentistry.

Dimensional change of alveolar bone following tooth loss has been one of the most highly conveyed clinical challenge documented in dentistry for over 50 years (Araújo & Lindhe, 2005; Chappuis et al., 2013; Johnson, 1963). Many reports over the past decade have attempted to minimize these effects via a variety of procedures including collagen barrier membranes, bone grafting materials and growth factors (Bayat, Momen Heravi, Mahmoudi, & Bahrami, 2015; Brkovic et al., 2008; Brkovic et al., 2012; Coomes et al., 2014; Fiorellini et al., 2005; Mardas, Chadha, & Donos, 2010; Mardas, D'Aiuto, Mezzomo, Arzoumanidi, & Donos, 2011; Misch, 2010; Wallace, 2015; Wallace, Pikos, & Prasad, 2014). Despite this, tooth loss remains a prominent challenge, and no single therapy has been shown capable of predictably preventing dimensional bone‐changes following extraction (De Risi, Clementini, Vittorini, Mannocci, & De Sanctis, 2015; Jambhekar, Kernen, & Bidra, 2015; Moraschini & Barboza, 2016; Morjaria, Wilson, & Palmer, 2014; Spagnoli & Choi, 2013; Tan, Wong, Wong, & Lang, 2012).

One low‐cost method utilized for the healing of extraction sockets by creating homeostasis is by placing an absorbable collagen sponge (ACS) into fresh sockets (Coomes et al., 2014). Advantages of ACS apart from their low‐cost include their stable and moldable cone‐shaped sizes, their natural collagen scaffolds, their ability to facilitate blood clot formation through hemostatic wound coverage, and their ability to remain rapidly and fully resorbable over time (Coomes et al., 2014). Although these properties are all contributing factors to extraction socket preservation and healing, one of their other advantages includes their ability to serve as a carrier system for growth factors due to their natural collagen structure (Coomes et al., 2014; De Sarkar et al., 2015; Spagnoli & Choi, 2013; Zhang et al., 2015).

In light of these advantages and due to the reported clinical challenges faced in daily clinical practice, the aim of the present study was to assess the regenerative potential of ACS loaded with rhBMP9. In a first step study, the regenerative potential of rhBMP9 was directly compared to rhBMP2 and investigated on in vitro adsorption and release of BMP9 from ACS. Thereafter, preosteoblasts were investigated for their ability to attach, proliferate, and differentiate onto ACS loaded with either rhBMP9 or rhBMP2 in two different concentrations each.

## 2 MATERIAL AND METHODS

### 2.1 Absorbable collagen sponges and rhBMP2 and rhBMP9

Recombinant human BMP2 and rhBMP9 were purchased from R&D systems Inc (Minneapolis, MM, USA). ACS, porcine‐origin native types I and III porcine collagen sponge, were kindly provided by Botiss AG, Germany (collacone®). Figure S1 demonstrates a scanning electron microscopy (SEM) image used to characterize surface shape and topography as previously described (Miron et al., 2011). For all in vitro experiments, the following six groups were used: (a) control standard tissue culture plastic (TCP), (b) control ACS alone, (c) ACS loaded with low concentration (10 ng/ml) of rhBMP2, (d)ACS loaded with high concentration (100 ng/ml) of rhBMP2, (e) ACS loaded with low concentration (10 ng/ml) of rhBMP9, and (f) ACS loaded with high concentration (100 ng/ml) of rhBMP9. Undifferentiated mouse cell‐line ST2 was obtained from RIKEN Cell Bank (Tsukuba, Japan) and therefore no ethical approval was necessary for the present study. Cells were cultured in a humidified atmosphere at 37 °C in growth medium consisting of Dulbecco's modified eagle's medium (DMEM; Invitrogen Corporation, Carlsbad, CA), 10% fetal Bovine serum (FBS; Invitrogen), and antibiotics (Invitrogen). For in vitro experiments, ACSs were cut in 1 mm thick cylinders to fit into the bottom of 24 well dishes and coated with rhBMP2 or rhBMP9 for 5 min prior to cell seeding. Thereafter, cells were seeded onto the various treatment modalities at a density of 10,000 cells in 24 well culture plates for cell adhesion and proliferation experiments and 50,000 cells per well in 24 well dishes for real‐time PCR, ALP assay, and alizarin red experiments. For experiments lasting longer than 5 days, medium was replaced twice weekly.

### 2.2 ELISA protein quantification of rhBMP2 and rhBMP9 adsorption to absorbable collagen sponges

To determine the quantity of rhBMP2 and rhBMP9 adsorption to the surface of ACS, enzyme‐linked immunosorbent assay (ELISA) quantification assay was utilized. Briefly, the coating period incubation of both 100 ng/mL of rhBMP2 and rhBMP9 onto ACS at 37 °C in a shaking incubator. The remaining Phosphate Buffered Solution (PBS), containing unattached protein, was collected and quantified by a sandwich ELISA, BMP2 (DY355, range = 46.90–3,000 pg/ml, R&D Systems, Minneapolis, MN, USA), and BMP9 (DY3209, range = 15.60–1,000 pg/ml, R&D Systems) for the amount of rhBMP2/rhBMP9 protein unadsorbed to ACS according to manufacturer's protocol. The 100 ng/ml of rhBMP2 and rhBMP9 in PBS was quantified by ELISA, and the amount of each protein was considered as positive control (total coated protein). Subtraction of total coated protein from the amount of unadsorbed protein was used to determine the amount of adsorbed material to the surface of ACS as previously described (Miron et al., 2015). Furthermore, in order to determine the quantity of rhBMP2 and rhBMP9 protein being released from ACS over time, coated ACS were soaked in 1 ml of PBS, and samples were collected at various time points including 15 min, 1 hr, 8 hrs, 1, 3, and 10 days. All samples were quantified in duplicate, and three independent experiments were performed.

### 2.3 Adhesion and proliferation assay

ST2 cells were seeded in 24 well plates at a density of 10,000 cells per well in either (a) control standard TCP, (b) control ACS alone, (c) have ACS loaded with low concentration (10 ng/ml) of rhBMP2, (d) have ACS loaded with high concentration (100 ng/ml) of rhBMP2, (e) have ACS loaded with low concentration (10 ng/ml) of rhBMP9, or (f) high concentration (100 ng/ml) of rhBMP9. Cells were quantified using MTS assay (3‐(4,5‐dimethylthiazol‐2‐yl)‐5‐(3‐carboxymethoxyphenyl)‐2‐(4‐sulfophenyl)‐2H‐tetrazolium) (Promega, Madison, WI) at 8 hrs for cell adhesion and at 1, 3, and 5 days for cell proliferation as previously described (Miron, Saulacic, Buser, Iizuka, & Sculean, 2013). At desired time points, cells were washed with PBS and quantified using an ELx808 Absorbance Reader (BIO‐TEK, Winooski, VT). Experiments were performed in triplicate with three independent experiments for each condition. Data were analyzed for statistical significance using one‐way analysis of variance for adhesion assay and two‐way analysis of variance with Turkey test for proliferation assay (*, *p* values <.05 was considered significant).

### 2.4 ALP activity assay

ST2 cells were stimulated on ACS with/without rhBMP in growth media. At 7 days, cells were quantified for ALP expression as determined cell imaging. ALP activity was monitored using leukocyte alkaline phosphatase kit (procedure No. 86, Sigma). ST2 cells were fixed by immersing in a citrate‐acetone‐formaldehyde fixative solution for 5 min and rinsed in deionized water for 1 min. Alkaline dye mixture are prepared by 1 ml Sodium Nitrite Solution and 1 ml fast red violet alkaline solution dissolved in 45 ml of distilled water and 1 ml of Naphtol AS‐Bl alkaline solution. Surfaces were then placed in alkaline dye mixture solution for 15 min protected from light. Following 2 min of rinsing in deionized water. All images were captured on a Wild Heerbrugg M400 ZOOM Makroskop (WILD HEERBRUGG, Switzerland) at the same magnification at the same light intensity and imported onto Image J software (NIH, Bethesda, MD). Thresholding was used to generate percent‐stained values for each field of view.

### 2.5 Real‐time PCR for osteoblast differentiation markers

Real‐time RT‐PCR was used to investigate the expression of genes encoding osteoblast differentiation markers. Total RNA was isolated using High Pure RNA Isolation Kit (Roche, Basel, Switzerland) at 3 and 14 days. Primer and probe sequences for genes encoding runt‐related transcription factor 2, collagen1α2 (COL1a2), ALP, bone sialoprotein (BSP), osteocalcin (OCN) and glyceraldehyde 3‐phosphate dehydrogenase were fabricated with Primer sequences according to Table 1. Reverse transcription was performed with Transcriptor First Strand cDNA Synthesis Kit (Roche). Real‐time RT‐PCR was performed using Roche FastStart Universal SYBR Green Master and quantified on an Applied Biosystems 7500 Real‐Time PCR Machine (Biosystems, Life Technologies Corporation, Carlsbad, CA). A Nanodrop 2000c (Thermo, Wilmington, DE) was used to quantify total RNA levels. All samples were assayed in duplicate with three independent experiments were performed. The ΔΔCt method was used to calculate gene expression levels normalized to mRNA level of glyceraldehyde 3‐phosphate dehydrogenase and calibrated to control samples. Data were analyzed for statistical significance using two‐way analysis of variance with Tukey test (*, *p* values <.05 was considered significant).

**Table 1:** Polymerase chain reaction primers for genes encoding runt‐related transcription factor, collagen 1 alpha 2, alkaline phosphatase, bone sialoprotein, osteocalcin, and glyceraldehyde 3‐phosphate dehydrogenase

Gene	Primer sequence
mRUNX2 F	Agggactatggcgtcaaaca
mRUNX2 R	Ggctcacgtcgctcatctt
mCOL1a2 F	Gagctggtgtaatgggtcct
mCOL1a2 R	Gagacccaggaagacctctg
mALP F	Ggacaggacacacacacaca
mALP R	Caaacaggagagccacttca
mBSP F	Gcactccaactgcccaaga
mBSP R	Ttttggagccctgctttctg
mOCN F	Cagacaccatgaggaccatc
mOCN R	Ggactgaggctctgtgaggt
mGAPDH F	Aggtcggtgtgaacggatttg
mGAPDH R	Tgtagaccatgtagttgaggtca

*Note*. Runx2 = encoding runt‐related transcription factor; COL1a2 = collagen 1 alpha 2; ALP = alkaline phosphatase; BSP = bone sialoprotein; OCN = osteocalcin; GAPDH = glyceraldehyde 3‐phosphate dehydrogenase

### 2.6 Alizarin red staining

ST2 cells were stimulated on ACS for 14 days with/without rhBMP in osteogenic differentiation medium, which consisted of DMEM supplemented with 10% FBS, 1% antibiotics, 50 μg/ml ascorbic acid (Sigma) and 10 mM β‐glycerophosphate (Sigma) to promote osteoblast differentiation. Alizarin red staining was performed to determine the presence of extracellular matrix mineralization. After 14 days, cells were fixed in 96% ethanol for 15 min and stained with 0.2% alizarin red (Alizarin red S; Sigma) solution in water (pH 6.4) at room temperature for 1 hr as previously described (Fujioka‐Kobayashi et al., 2016a). Alizarin red quantification was performed using images captured on a Nikon D610 camera with a Heerbrugg M400 ZOOM microscope (Wild Heerbrugg, Switzerland). The same threshold values were used for all analyzed. Means and standard errors were calculated, and the statistical significance of differences was examined by one‐way analysis with Tukey test between both groups (*, *p* values <.05 was considered significant).

## 3 RESULTS

### 3.1 Surface characteristics of absorbable collagen sponges and ability to adsorb and release rhBMP2 and rhBMP9 over time

**Figure 1:**
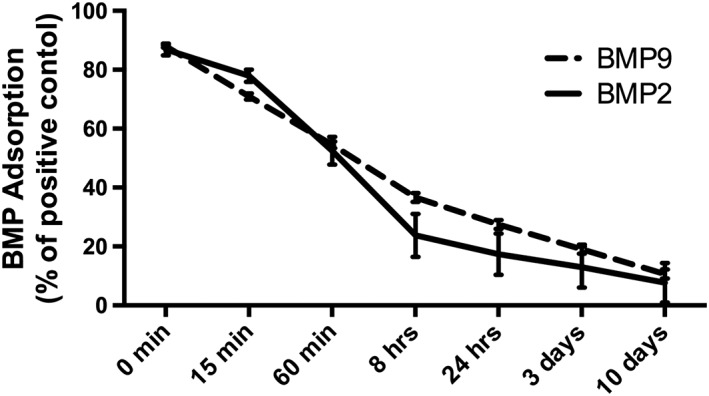
Growth factor adsorption of recombinant human bone morphogenetic protein 2 and recombinant human bone morphogenetic protein 9to absorbable collagen sponges at 0 min, 15 min, 60 min, 8 hrs, 24 hrs, 3 days, and 10 days as quantified by enzyme‐linked immunosorbent assay. absorbable collagen sponges were able to efficiently adsorb recombinant bone morphogenetic protein 2 and bone morphogenetic protein 9 and have comparable release kinetics over time

First, the surface morphology and characteristics of ACS were investigated via low and high magnification SEM (Figure S1). It was found at low magnification that the three‐dimensional surface architecture of ACS resembled a “honeycomb”‐shaped morphology fabricated from collagen fibrils (Figure S1). The high‐resolution SEM images further demonstrated the numerous collagen fibers found on the surface of ACS scaffolds (Figure S1). Thereafter, the adsorption and release kinetics of both rhBMP2 and rhBMP9 were investigated at various time points ranging from zero to 10 days (Figure 1). ACSs were able to adsorb nearly 90% of total protein content of rhBMP2 and rhBMP9. Over time, ACS displayed a feature of slowly dissolving and releasing rhBMP2 or rhBMP9 over a 10‐day (Figure 1). No differences in growth factor adsorption could be observed between rhBMP2 and rhBMP9 (Figure 1). At the end of the experiment (10 days), approximately 20% of the initial growth factor concentration remained present within the collagen scaffolds demonstrating a slow and consistent release of either rhBMP2 or rhBMP9 over time (Figure 1).

### 3.2 Effect of absorbable collagen sponges on ST2 cell adhesion and proliferation when combined with rhBMP2 or rhBMP9

**Figure 2:**
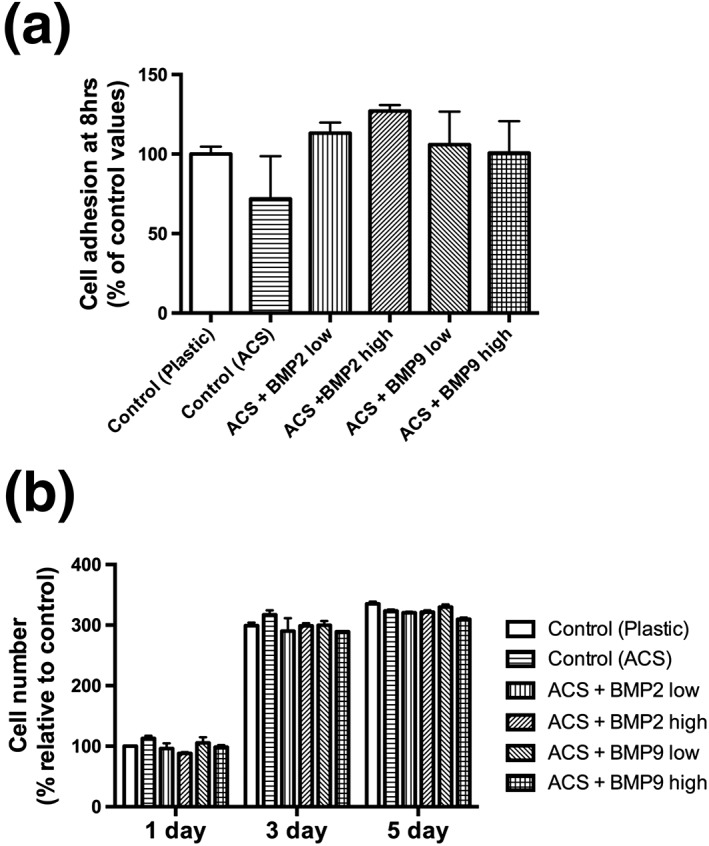
(a) Attachment (8 hrs) and (b) proliferation (1, 3, and 5 days) assays of ST2 cells seeded on control tissue culture plastic, control absorbable collagen sponges (ACS), ACS loaded with bone morphogenetic protein 2 low (10 ng/ml), ACS loaded with bone morphogenetic protein 2 high (100 ng/ml), ACS loaded with bone morphogenetic protein 9 low (10 ng/ml), and ACS loaded with bone morphogenetic protein 9 high (100 ng/ml). No significant difference was observed between six groups at all time points

Thereafter, ST2 preosteoblasts were seeded directly onto ACS and investigated for cell adhesion at 8 hrs (Figure 2a) and cell proliferation at 1, 3 and 5 days postseeding (Figure 2b). It was first found that cells attached efficiently to ACS in a near 100% value when compared to standard TCP (Figure 2a). No differences could be observed between control TCP, control ACS as well as any of the groups containing rhBMP2 or rhBMP9 (Figure 2a). Furthermore, the effects of rhBMP2 and rhBMP9 had no influence on the proliferation of preosteoblasts at either 1, 3, or 5 days postseeding (Figure 2b).

### 3.3 Effect of absorbable collagen sponges on ST2 cell differentiation when combined with rhBMP2 or rhBMP9

**Figure 3:**
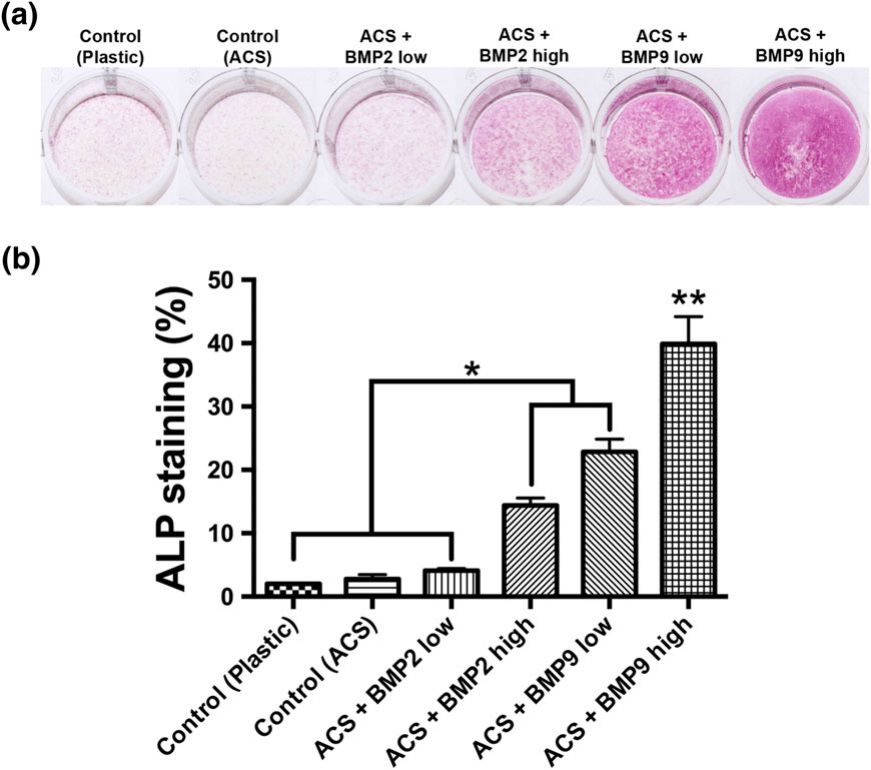
(a) Alkaline phosphatase staining of absorbable collagen sponges (ACS) at 7 days of ST2 cells seeded on control tissue culture plastic, control ACS, ACS loaded with BMP2 low (10 ng/ml), ACS loaded with BMP2 high (100 ng/ml), ACS loaded with BMP9 low (10 ng/ml), and ACS loaded with BMP9 high (100 ng/ml). (b) bone morphogenetic protein 9 low and high significantly increased alkaline phosphatase staining when compared to control and bone morphogenetic protein 2samples (* denotes significant difference, *p* < .05; ** denotes significantly higher than all other treatment modalities, *p* < .05)

**Figure 4:**
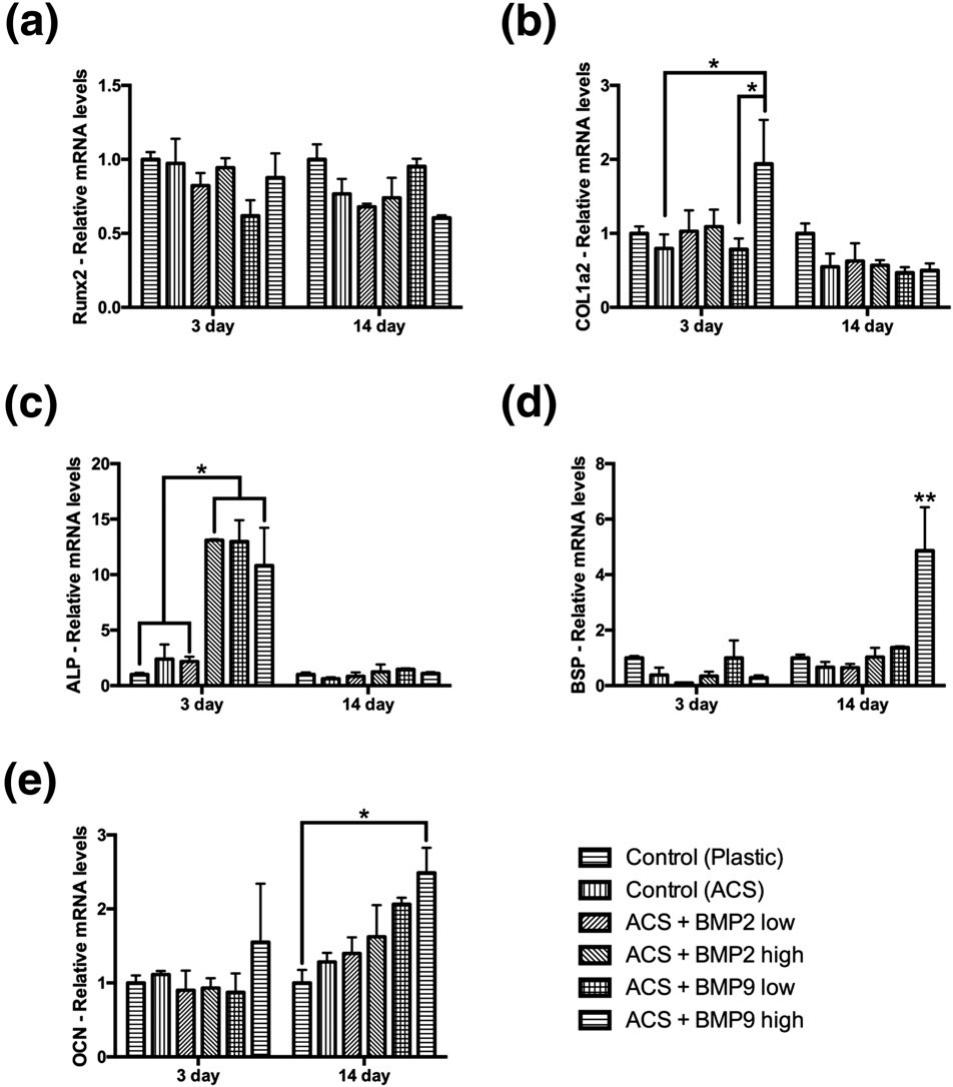
Real‐time PCR of ST2 cells seeded on control tissue culture plastic, control absorbable collagen sponges (ACS), ACS loaded with BMP2 low (10 ng/ml), ACS loaded with BMP2 high (100 ng/ml), ACS loaded with BMP9 low (10 ng/ml), and ACS loaded with BMP9 high (100 ng/ml) for genes encoding (a) runt‐related transcription factor 2, (b) collagen 1 alpha 2 (COL1a2), (c) alkaline phosphatase (ALP), (d) bone sialoprotein (BSP), and (e) osteocalcin (OCN) at 3 and 14 days postseeding (* denotes significant difference, *p* < .05; ** denotes significantly higher than all other treatment modalities, *p* < .05)

**Figure 5:**
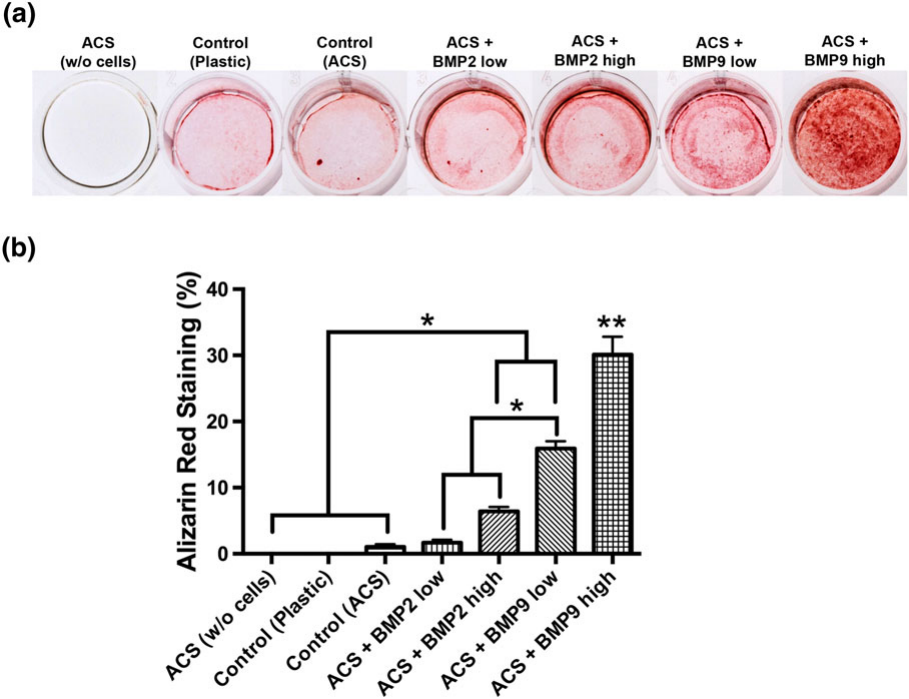
(a) Visual representation of alizarin red‐stained of negative control absorbable collagen sponges (ACS) without cells, control tissue culture plastic, control ACS, ACS loaded with BMP2 low (10 ng/ml), ACS loaded with BMP2 high (100 ng/ml), ACS loaded with BMP9 low (10 ng/ml), and ACS loaded with BMP9 high (100 ng/ml) at 14 days postseeding. Note the intensity of red staining of ACS coated with BMP9 in comparison to control and BMP2 samples. (b) Quantified data of alizarin red staining from colour thresholding software (* denotes significant difference, *p* < .05; ** denotes significantly higher than all other treatment modalities, *p* < .05)

The effects of rhBMP2 and rhBMP9 were investigated on ST2 cell differentiation to osteoblasts (Figure 3, 4, 5). It was observed that ACS coated with either rhBMP2 high or rhBMP9 (low or high) demonstrated significantly higher ALP staining when compared to control TCP, control ACS, and ACS coated with rhBMP2 low at a concentration of 10 ng/ml. The ACS loaded with rhBMP2 high (100 ng/ml) demonstrated up to a five‐fold increase in ALP staining when compared to controls (Figure 3). Interestingly, rhBMP9 low demonstrated a 1.5‐fold increase when compared to rhBMP2 high (representing a one‐fold lower concentration of rhBMP9 low (10 ng/ml) when compared to rhBMP2 high (100 ng/ml)). ACS loaded with rhBMP9 high (100 ng/ml) demonstrated significantly higher ALP staining when compared to all other groups representing up to a 20‐fold significant increase when compared to control samples and a 2.5 fold significant increase when compared to rhBMP2 at the same concentration (100 ng/ml; Figure 3).

Thereafter, real‐time PCR was utilized to investigate osteoblast differentiation markers at 3 and 14 days post seeding (Figure 4). While runt‐related transcription factor 2 demonstrated no differences between all groups at either time point (Figure 4a), COL1a2 demonstrated significantly higher levels of rhBMP9 high when compared to control ACS at 3 days (Figure 4b). The mRNA levels of ALP further demonstrated significantly higher values for rhBMP9 low, rhBMP9 high and rhBMP2 high when compared to control TCP, control ACS alone, and ACS loaded with rhBMP2 low (Figure 4c). No differences could be observed between any groups by 14 days (Figure 4c). Late osteoblast differentiation markers including BSP and OCN both demonstrated significantly higher levels in the rhBMP9 high group at 14 days postseeding (Figure 4d,e).

In a final experiment, alizarin red staining was utilized to investigate the mineralization potential of each group over a 14 day period (Figure 5). It was observed that the groups preloaded with rhBMP9 performed significantly better than rhBMP2 and control groups (Figure 5). rhBMP9 low (10 ng/ml) demonstrated over a two‐fold increase in alizarin red staining when compared to rhBMP2 high (100 ng/ml) (Figure 5). Furthermore, rhBMP9 high demonstrated up to a four‐fold increase when compared to rhBMP2 high (Figure 5).

## 4 DISCUSSION

The aim of the present study was to investigate the biocompatibility of ACS and their ability to promote osteoblast differentiation when combined with rhBMP2 and rhBMP9. The use of ACS shaped in cylindrical format has been utilized in modern dentistry to facilitate wound healing in extraction sockets. Although numerous scientific reports have now documented the effects of tooth loss on dimensional changes of alveolar bone (Araújo & Lindhe, 2005; Chappuis et al., 2013; De Risi et al., 2015; Jambhekar et al., 2015; Moraschini & Barboza, 2016; Morjaria et al., 2014; Spagnoli & Choi, 2013; Tan et al., 2012), there remains a lack of options to predictably regenerate lost bone following tooth loss. For these reasons, a variety of treatment options and various biomaterials have been investigated to minimize bone loss following tooth extraction (Bayat et al., 2015; Brkovic et al., 2008; Brkovic et al., 2012; Coomes et al., 2014; Fiorellini et al., 2005; Mardas et al., 2010; Mardas et al., 2011; Misch, 2010; Wallace, 2015; Wallace et al., 2014).

One available option for regenerating missing bone is the use of BMPs which has been exploited as a recombinant growth factor source for a variety of bone augmentation procedures including guided bone regeneration, sinus augmentation, and vertical and horizontal augmentation procedures (Draenert, Nonnenmacher, Kammerer, Goldschmitt, & Wagner, 2013; Fiorellini et al., 2005; Leknes et al., 2008; Schwarz et al., 2008). Numerous previous research have further demonstrated that the optimal loading carrier system for recombinant growth factors continues to be the use of scaffolds containing collagen matrix such as ACS (Coomes et al., 2014; De Sarkar et al., 2015; Spagnoli & Choi, 2013; Zhang et al., 2015).

In the present study, it was found via SEM that these ACS had a very porous surface structure demonstrating a honeycomb‐shaped surface morphology at low magnificent; important features for growth factor adsorption as well as penetration of progenitor cells into the material surface (Figure S1). Furthermore, the growth factor adsorption properties and release kinetics further demonstrated the favorable loading of either rhBMP2 or rhBMP9 and the ability for the biomaterial to hold and release the growth factor over a 10 day period (Figure 1). The ability for growth factors to adsorb better to ACS when compared to mineralized bone grafting particles has previously been investigated by our group (Miron et al., 2015). Along with unpublished data from our laboratory, it was also found that rhBMP9 adsorbed more efficiently to ACS when compared to a variety of other bone grafting particles including demineralized freeze dried bone allograft, a natural bone mineral of bovine origin and calcium phosphate biomaterials.

One of the major challenges faced to date with the use of recombinant BMP2 is the number of reported secondary side effects including less than optimal bone volume and density, edema, and inflammation have been associated with their high dosage (Tannoury & An, 2014). For these reasons, more efficient growth factors that may be utilized in lower doses would present a major breakthrough in regenerative medicine in the bone biology fields.

Recently, our group found that rhBMP9 induced significantly higher osteoblast differentiation potential when compared to rhBMP2. In the present investigation, we utilized these preliminary findings and attempt to optimize the results by combining rhBMP9 with ACS scaffolds. In the present study, we found that rhBMP9 even at low concentrations of 10 ng/ml was able to significantly induce over a two‐fold increase in osteoblast differentiation when compared to rhBMP2 at a high concentration of 100 ng/ml. Furthermore, positive results were also observed for experiments investigating mRNA levels of osteoblast differentiation markers as well as mineralization potential investigated via alizarin red staining. Therefore, it may be hypothesized that based on these positive results, lower concentrations of rhBMP9 could potentially be utilized to induce new bone formation when compared to rhBMP2. The difference in osteogenic behavior between BMP2 and BMP9 was addressed previously by several pathway variances according to adenovirus experiments. BMP antagonist, noggin inhibited BMP2 osteogenesis, however SMAD phosphorylation by BMP9 was not inhibited by exogenous noggin (Bergeron et al., 2009; Nakamura, Shinohara, Momozaki, Yoshimoto, & Noguchi, 2013; Wang et al., 2013). BMP3, a known BMP2 inhibitor, did not inhibit BMP9‐mediated bone formation (Kang et al., 2004). Further research utilizing rhBMP9 would benefit our understanding on the differential mechanisms that regulate osteogenesis in both BMP2 versus BMP9. One group currently reported that rhBMP9 loaded on atelocollagen and chitosan sponges promoted new bone formation in rat critical‐sized bone defect (Toshiaki Nakamura et al., 2016; Shinohara, Nakamura, Shirakata, & Noguchi, 2016). The further animal studies in comparison with rhBMP2 are now necessary to further verify bone regenerative potential of rhBMP9.

The findings from the present study demonstrate that ACS loaded with either rhBMP2 or rhBMP9 were able to efficiently adsorb both growth factors onto ACS over a 10‐day period and induce osteoblast differentiation. Noteworthy, rhBMP9 was able to stimulate over a two‐fold increase in osteoblast differentiation at low doses (10 ng/ml) when compared to rhBMP2 at high doses (100 ng/ml). Future animal studies are now needed to investigate the regenerative potential of rhBMP9 in combination with ACS to minimize dimensional changes in extraction sockets following tooth loss.

## CONFLICT OF INTEREST

All authors affirm that they have no conflict of interest.

## Supporting information



Figure S1. Scanning electron microscopy (SEM) of absorbable collagen sponges (ACS) at (A) low and (B) high magnification. Notice the honeycomb shaped morphology of the collagen scaffolds with numerous pores.
