# Supplementary figures and images for: Absorbable collagen sponges loaded with recombinant bone morphogenetic protein 9 induces greater osteoblast differentiation when compared to bone morphogenetic protein 2
Source: Clin Exp Dent Res. 2017 Feb 9;3(1):32–40. doi: 10.1002/cre2.55 (PMC5839213; doi:10.1002/cre2.55)

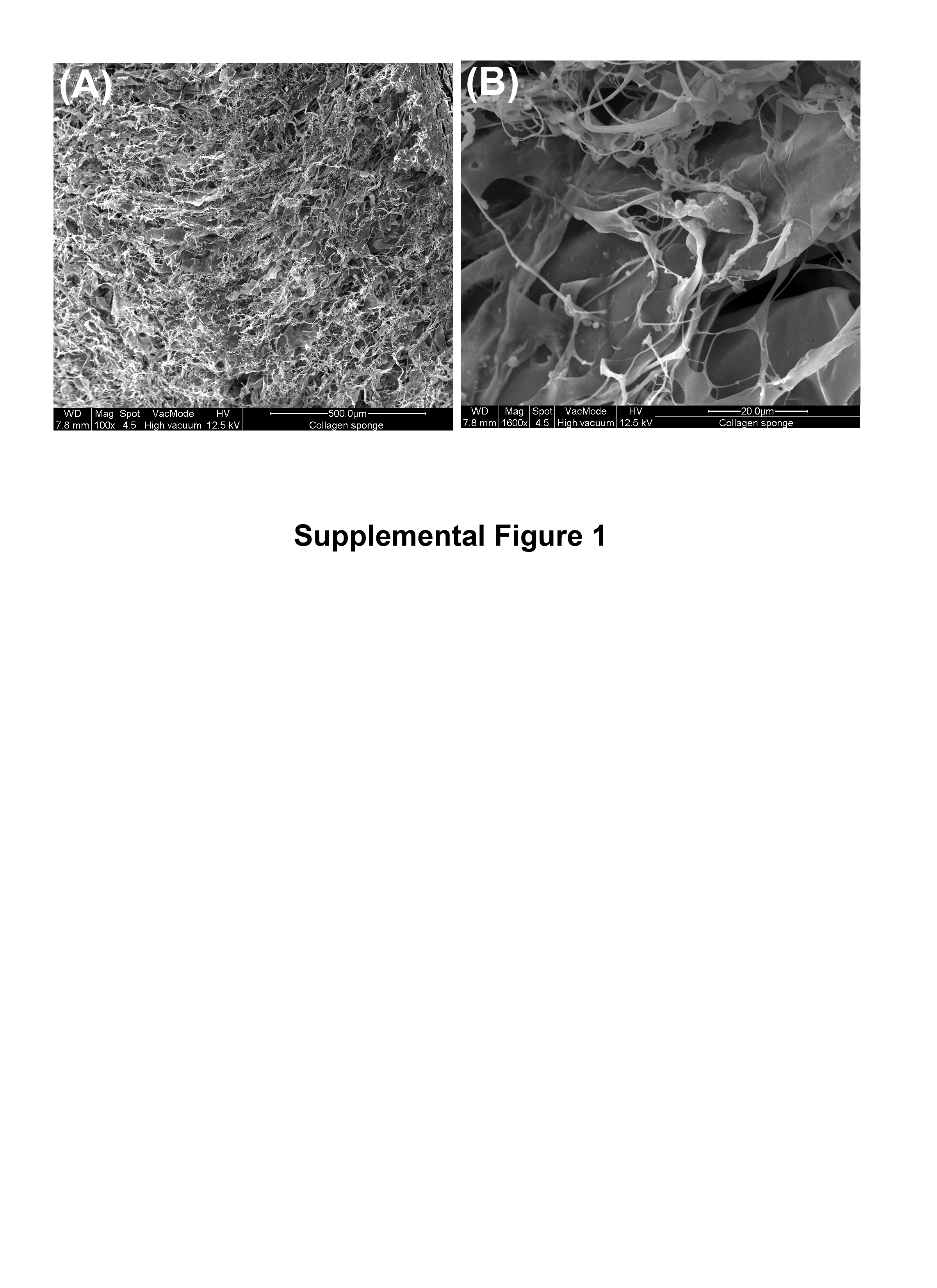

Supplement: Supplementary file 1 — Figure S1. Scanning electron microscopy (SEM) of absorbable collagen sponges (ACS) at (A) low and (B) high magnification. Notice the honeycomb shaped morphology of the collagen scaffolds with numerous pores. [file CRE2-3-32-s001.tif]
